# (Acetonitrile-κ*N*)penta­carbonyl­tungsten(0)

**DOI:** 10.1107/S1600536811017879

**Published:** 2011-05-20

**Authors:** Christoph E. Strasser, Stephanie Cronje, Helgard G. Raubenheimer

**Affiliations:** a Department of Chemistry and Polymer Science, University of Stellenbosch, Private Bag X1, Matieland 7602, South Africa

## Abstract

The acetonitrile ligand in the title compound, [W(CH_3_CN)(CO)_5_], is coordinated end-on to a penta­carbonyl­tungsten(0) fragment with a W—N bond length of 2.186 (4) Å, completing an octa­hedral coordination environment around the W atom.

## Related literature

For other structures with an (alkyl nitrile-κ*N*)penta­carbonyl­tungsten(0) fragment, see: Darensbourg *et al.* (1992 ▶); Reibenspies *et al.* (1994 ▶); Jefford *et al.* (1996 ▶). For structures with conjugated nitriles, see: Fischer *et al.* (1993 ▶); Helten *et al.* (2010 ▶) and for structures with nitriles that are part of an organometallic complex, see: Busetto *et al.* (1992 ▶); Duclos *et al.* (1999 ▶); Tang *et al.* (1999 ▶); Trylus *et al.* (1999 ▶); Cordiner *et al.* (2006 ▶). For the preparation, see: Strasser *et al.* (2010 ▶).
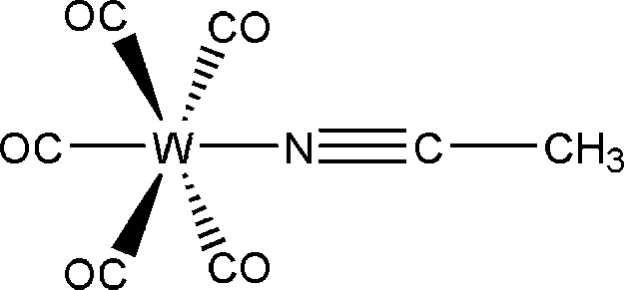


## Experimental

### 

#### Crystal data

[W(C_2_H_3_N)(CO)_5_]
*M* *_r_* = 364.95
Monoclinic, 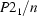

*a* = 5.6485 (6) Å
*b* = 13.6231 (15) Å
*c* = 12.8642 (15) Å
β = 101.883 (2)°
*V* = 968.69 (19) Å^3^
*Z* = 4
Mo *K*α radiation
μ = 11.92 mm^−1^
*T* = 100 K
0.17 × 0.07 × 0.05 mm

#### Data collection

Bruker APEX CCD area-detector diffractometer
Absorption correction: multi-scan (*SADABS*; Bruker, 2002 ▶) *T*_min_ = 0.337, *T*_max_ = 0.549
5297 measured reflections
1973 independent reflections
1694 reflections with *I* > 2σ(*I*)
*R*_int_ = 0.021

#### Refinement

*R*[*F* ^2^ > 2σ(*F* ^2^)] = 0.022
*wR*(*F* ^2^) = 0.055
*S* = 1.08
1973 reflections
128 parameters
H-atom parameters constrained
Δρ_max_ = 1.69 e Å^−3^
Δρ_min_ = −1.08 e Å^−3^

### 

Data collection: *SMART* (Bruker, 2002 ▶); cell refinement: *SAINT* (Bruker, 2003 ▶); data reduction: *SAINT*; program(s) used to solve structure: *SHELXS97* (Sheldrick, 2008 ▶); program(s) used to refine structure: *SHELXL97* (Sheldrick, 2008 ▶); molecular graphics: *X-SEED* (Atwood & Barbour, 2003; ▶ Barbour, 2001 ▶); software used to prepare material for publication: *X-SEED*.

## Supplementary Material

Crystal structure: contains datablocks I, global. DOI: 10.1107/S1600536811017879/ds2110sup1.cif

Structure factors: contains datablocks I. DOI: 10.1107/S1600536811017879/ds2110Isup2.hkl

Additional supplementary materials:  crystallographic information; 3D view; checkCIF report

## Figures and Tables

**Fig. 1.**
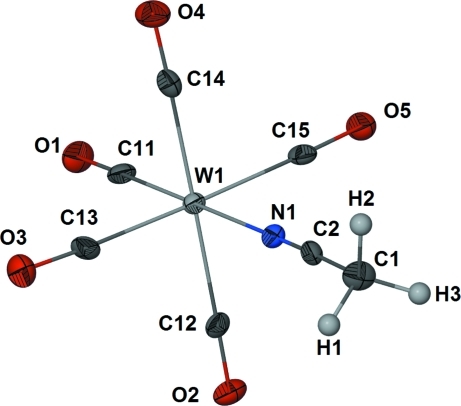
The asymmetric unit of the title compound, ellipsoids are drawn at the 50% probability level.

## supplementary crystallographic information

### Comment

The title compound is an important starting material in the synthesis of complexes containing the pentacarbonyltungsten(0) fragment. The nitrile ligand is coordinated end-on to W(CO)_5_, completing an octahedral coordination geometry at the tungsten atom.

The *trans*-influence of the acetonitrile ligand is echoed by the comparatively short W1—C11 bond [1.975 (5) Å] compared to the *cis*-carbonyls [W—C bond lengths 2.023 (5) Å to 2.056 (5) Å].

When the present molecular structure of [W(CH_3_CN)(CO)_5_] is compared to other literature examples of (nitrile-κ*N*)pentacarbonyltungsten(0) compounds, surprisingly no significant variations in bond lengths can be observed within the metal–ligand bonds despite different electronic and steric properties of the nitriles used. Related crystal structures reported include the W(CO)_5_ adducts of alkylnitriles (Reibenspies *et al.*, 1994; Jefford *et al.*, 1996), conjugated nitriles (Fischer *et al.*, 1993; Tang *et al.*, 1999; Cordiner *et al.*, 2006; Helten *et al.*, 2010), α-metal-substituted alkyl nitriles (Busetto *et al.*, 1992; Trylus *et al.*, 1999) as well as deprotonated 2,3,4-tricyanopent-2-enedinitrile (Duclos *et al.*, 1999).

### Experimental

The compound was obtained as a side product after chromatography of a mixture obtained by treating pentacarbonyl[(4-methyl-1,3-thiazol- 2-yl)carbonyl]tungsten(0) with bis(trichloromethyl)carbonate (Strasser *et al.*, 2010). Decomposition of the initial product followed by reaction of the pentacarbonyltungsten fragment with acetonitrile in the mobile phase resulted in formation of the title compound. Crystals of the title compound were formed by layering a dichloromethane solution with hexane.

### Refinement

All H atoms were positioned geometrically (C—H = 0.98 Å) and constrained to ride on their parent atoms; the *U*_iso_(H) values were set at 1.5 times *U*_eq_(C).

The maximum residual electron density of 1.69 e Å^-3^ is located 0.85 Å next to W1.

### Crystal data

**Table:**

[W(C_2_H_3_N)(CO)_5_]	*F*(000) = 664
*M**_r_* = 364.95	*D*_x_ = 2.502 Mg m^−^^3^
Monoclinic, *P*2_1_/*n*	Mo *K*α radiation, λ = 0.71073 Å
Hall symbol: -P 2yn	Cell parameters from 3152 reflections
*a* = 5.6485 (6) Å	θ = 2.2–26.4°
*b* = 13.6231 (15) Å	µ = 11.92 mm^−^^1^
*c* = 12.8642 (15) Å	*T* = 100 K
β = 101.883 (2)°	Prism, light yellow
*V* = 968.69 (19) Å^3^	0.17 × 0.07 × 0.05 mm
*Z* = 4	

### Data collection

**Table:**

Bruker APEX CCD area-detector diffractometer	1973 independent reflections
Radiation source: fine-focus sealed tube	1694 reflections with *I* > 2σ(*I*)
graphite	*R*_int_ = 0.021
ω scans	θ_max_ = 26.4°, θ_min_ = 2.2°
Absorption correction: multi-scan (*SADABS*; Bruker, 2002)	*h* = −7→7
*T*_min_ = 0.337, *T*_max_ = 0.549	*k* = −17→16
5297 measured reflections	*l* = −16→10

### Refinement

**Table:**

Refinement on *F*^2^	Primary atom site location: structure-invariant direct methods
Least-squares matrix: full	Secondary atom site location: difference Fourier map
*R*[*F*^2^ > 2σ(*F*^2^)] = 0.022	Hydrogen site location: inferred from neighbouring sites
*wR*(*F*^2^) = 0.055	H-atom parameters constrained
*S* = 1.08	*w* = 1/[σ^2^(*F*_o_^2^) + (0.0282*P*)^2^ + 0.8554*P*] where *P* = (*F*_o_^2^ + 2*F*_c_^2^)/3
1973 reflections	(Δ/σ)_max_ = 0.002
128 parameters	Δρ_max_ = 1.69 e Å^−^^3^
0 restraints	Δρ_min_ = −1.08 e Å^−^^3^

### Special details

**Table:**

Geometry. All e.s.d.'s (except the e.s.d. in the dihedral angle between two l.s. planes) are estimated using the full covariance matrix. The cell e.s.d.'s are taken into account individually in the estimation of e.s.d.'s in distances, angles and torsion angles; correlations between e.s.d.'s in cell parameters are only used when they are defined by crystal symmetry. An approximate (isotropic) treatment of cell e.s.d.'s is used for estimating e.s.d.'s involving l.s. planes.
Refinement. Refinement of *F*^2^ against ALL reflections. The weighted *R*-factor *wR* and goodness of fit *S* are based on *F*^2^, conventional *R*-factors *R* are based on *F*, with *F* set to zero for negative *F*^2^. The threshold expression of *F*^2^ > 2σ(*F*^2^) is used only for calculating *R*-factors(gt) *etc*. and is not relevant to the choice of reflections for refinement. *R*-factors based on *F*^2^ are statistically about twice as large as those based on *F*, and *R*- factors based on ALL data will be even larger.

### Fractional atomic coordinates and isotropic or equivalent isotropic displacement parameters (Å^2^)

**Table:**

	*x*	*y*	*z*	*U*_iso_*/*U*_eq_	
W1	0.12706 (3)	0.623964 (12)	0.793281 (13)	0.01429 (8)	
O1	0.3950 (6)	0.4656 (3)	0.6833 (3)	0.0283 (8)	
O2	−0.1535 (6)	0.4537 (2)	0.8890 (3)	0.0258 (8)	
O3	−0.2754 (7)	0.6294 (2)	0.5820 (3)	0.0296 (8)	
O4	0.4077 (6)	0.7892 (3)	0.6922 (3)	0.0304 (9)	
O5	0.5558 (7)	0.6182 (2)	0.9985 (3)	0.0263 (8)	
N1	−0.0645 (7)	0.7352 (3)	0.8667 (3)	0.0172 (8)	
C1	−0.3065 (9)	0.8698 (3)	0.9416 (4)	0.0247 (11)	
H1	−0.4783	0.8629	0.9087	0.037*	
H2	−0.2493	0.9347	0.9252	0.037*	
H3	−0.2848	0.8623	1.0188	0.037*	
C2	−0.1690 (8)	0.7948 (3)	0.9004 (4)	0.0171 (9)	
C11	0.2982 (8)	0.5241 (3)	0.7252 (4)	0.0199 (10)	
C12	−0.0548 (8)	0.5153 (3)	0.8553 (4)	0.0183 (9)	
C13	−0.1353 (8)	0.6289 (3)	0.6600 (4)	0.0202 (10)	
C14	0.3031 (9)	0.7309 (3)	0.7303 (4)	0.0205 (10)	
C15	0.4008 (8)	0.6203 (3)	0.9262 (4)	0.0191 (10)	

### Atomic displacement parameters (Å^2^)

**Table:**

	*U*^11^	*U*^22^	*U*^33^	*U*^12^	*U*^13^	*U*^23^
W1	0.01444 (11)	0.01441 (11)	0.01459 (11)	−0.00045 (6)	0.00428 (8)	−0.00013 (7)
O1	0.029 (2)	0.0272 (19)	0.032 (2)	0.0021 (15)	0.0142 (17)	−0.0088 (16)
O2	0.0288 (19)	0.0226 (17)	0.030 (2)	−0.0014 (14)	0.0161 (17)	0.0021 (15)
O3	0.0275 (19)	0.036 (2)	0.0238 (19)	−0.0018 (16)	0.0005 (17)	−0.0008 (16)
O4	0.0265 (19)	0.033 (2)	0.031 (2)	−0.0066 (16)	0.0033 (17)	0.0104 (16)
O5	0.0228 (18)	0.032 (2)	0.0234 (19)	−0.0028 (14)	0.0031 (16)	0.0037 (15)
N1	0.0175 (19)	0.0197 (19)	0.0147 (19)	−0.0014 (15)	0.0040 (16)	0.0010 (16)
C1	0.022 (2)	0.022 (2)	0.033 (3)	0.0035 (19)	0.011 (2)	−0.004 (2)
C2	0.018 (2)	0.018 (2)	0.014 (2)	−0.0032 (18)	0.0008 (19)	−0.0012 (18)
C11	0.017 (2)	0.022 (2)	0.024 (3)	−0.0038 (19)	0.011 (2)	−0.001 (2)
C12	0.020 (2)	0.019 (2)	0.017 (2)	0.0008 (18)	0.0091 (19)	−0.0029 (19)
C13	0.010 (2)	0.025 (2)	0.025 (3)	0.0002 (18)	0.0033 (19)	−0.002 (2)
C14	0.018 (2)	0.022 (2)	0.019 (2)	0.000 (2)	−0.001 (2)	−0.002 (2)
C15	0.018 (2)	0.019 (2)	0.022 (2)	−0.0039 (18)	0.007 (2)	0.0045 (19)

### Geometric parameters (Å, °)

**Table:**

W1—N1	2.186 (4)	C1—H1	0.9800
W1—C11	1.975 (5)	C1—H2	0.9800
W1—C12	2.054 (5)	C1—H3	0.9800
W1—C13	2.023 (5)	C11—O1	1.160 (6)
W1—C14	2.024 (5)	C12—O2	1.141 (6)
W1—C15	2.056 (5)	C13—O3	1.143 (6)
N1—C2	1.140 (6)	C14—O4	1.157 (6)
C1—C2	1.448 (6)	C15—O5	1.139 (6)
			
O1—C11—W1	178.6 (5)	C11—W1—C14	89.6 (2)
O2—C12—W1	178.8 (4)	C11—W1—C15	90.0 (2)
O3—C13—W1	176.5 (4)	C12—W1—C15	90.58 (18)
O4—C14—W1	177.3 (5)	C13—W1—C12	90.86 (19)
O5—C15—W1	178.6 (4)	C13—W1—C14	88.38 (19)
N1—C2—C1	178.7 (5)	C13—W1—C15	178.36 (16)
C2—N1—W1	176.9 (4)	C14—W1—C12	179.23 (19)
C11—W1—N1	179.26 (18)	C14—W1—C15	90.18 (18)
C12—W1—N1	90.02 (16)	C2—C1—H1	109.5
C13—W1—N1	90.09 (16)	C2—C1—H2	109.5
C14—W1—N1	90.09 (18)	C2—C1—H3	109.5
C15—W1—N1	90.70 (16)	H1—C1—H2	109.5
C11—W1—C13	89.2 (2)	H1—C1—H3	109.5
C11—W1—C12	90.32 (19)	H2—C1—H3	109.5
